# Concerns, attitudes, beliefs and information seeking practices with respect to nutrition-related issues: a qualitative study in French pregnant women

**DOI:** 10.1186/s12884-016-1078-6

**Published:** 2016-10-12

**Authors:** Clélia M. Bianchi, Jean-François Huneau, Gaëlle Le Goff, Eric O. Verger, François Mariotti, Patricia Gurviez

**Affiliations:** 1 UMR Physiologie de la Nutrition et du Comportement Alimentaire, AgroParisTech, INRA, Université Paris-Saclay, 16, rue Claude Bernard, 75005 Paris, France; 2 UMR Ingénierie Procédés Aliments, AgroParisTech, INRA, Université Paris-Saclay, 1, avenue des Olympiades, 91300 Massy, France; 3 IRD (Institut de Recherche pour le Développement), UMR NUTRIPASS IRD-UM-SupAgro, 34000 Montpellier, France

**Keywords:** Pregnancy, Maternal nutrition, Qualitative methods, Eating behaviour, Dietary modifications, Information behaviour

## Abstract

**Background:**

From a life course perspective, pregnancy leads to a rise in nutrition awareness and an increase in information flow in favour of adopting healthier eating behaviours. This qualitative study was designed to better understand the determinants of eating behaviours in French pregnant women by focusing on their concerns, attitudes and beliefs and their nutrition-related information seeking practices.

**Methods:**

Seven focus groups were conducted, involving a total of 40 French pregnant women. An inductive thematic approach, adapted from the grounded theory, was adopted to analyse the data. Two major themes were identified: eating behaviour and nutrition-related information behaviour.

**Results:**

The eating behaviour theme was divided into four sub-themes using the attribution theory. Three external causes affected the eating behaviour of pregnant women (food restrictions, physiological changes and weight gain), and led to frustration and a perceived loss of control. By contrast the adoption of a healthier diet was perceived as internal by pregnant women, and resulted in self-fulfilment and empowerment regarding the health and the well-being of their baby and themselves, and their weight gain management. Greater attention was paid to nutrition-related information obtained from healthcare providers, the social environment and the mass media. Information was passively absorbed or actively sought by pregnant women, but most was perceived as contradictory, which led to confusion.

**Conclusion:**

Pregnancy is accompanied by a rise in nutrition awareness, substantiated by eating behaviour modifications due to external and internal causes. However, conflicts between and within information sources result in confusion that can limit the adoption of healthier eating behaviour.

**Electronic supplementary material:**

The online version of this article (doi:10.1186/s12884-016-1078-6) contains supplementary material, which is available to authorized users.

## Background

According to the life course perspective, there are critical periods in an individual’s life, related to nutrition and health behaviours, that may cause specific and lasting changes to lifestyle trajectories [1, 2]. These trajectories, modelled across life by an aggregation of cultural, contextual and social factors, remain quite stable and difficult to modify, except during some transitional periods [3]. Pregnancy represents a ‘transition’ or ‘turning point’ in a woman’s life when biological, physiological, social and emotional changes are experienced [1, 2, 4]. Therefore, during the periconceptional period, women may become keener to engage in global and nutrition-related healthier behaviours which might be sustained over time and positively affect both their future health and that of their family. Within the framework of the life course perspective, previous studies described a rise in nutrition awareness during the periconceptional period [5, 6]. The rise in nutrition awareness also exists in second-time pregnant women but is less intense, because the healthier dietary patterns they adopted during their first pregnancy have at least partially been integrated in their food habits [6]. The acquisition of information is a crucial step when adopting healthier behaviour [7]. Greater nutrition awareness is accompanied by an increase in nutrition-related information seeking behaviour [8, 9]. Information can be obtained from healthcare professionals, by benefiting from other people’s experiences in their social environment or via the media [8]. Healthcare providers are most likely to offer a good response to pregnant women seeking nutrition-related information, but the quality of antenatal dietary information varies markedly, depending on the professional’s experience and time [10], so pregnant women often seek for, and find, information by themselves [8, 11]. Understanding how behaviour towards nutrition-related information affects the eating behaviour of pregnant women remains critical. Because of growing evidence that adequate maternal nutrition is critical to the health of both the mother and her offspring [12–16] and may even impact that of the child’s in the future [17–21], eating behaviours during pregnancy have recently been described in the nutrition research literature using qualitative methods. It nevertheless remains an under-investigated area. Until now, most qualitative studies focused on pregnant women’s weight gain [4, 22–27], especially among overweight and obese women [22, 23, 26, 27]. Their results confirmed the rise in nutrition awareness that occurs during pregnancy to be a healthy mother with limited weight gain and a healthy baby. Pregnant women adopted a healthier diet by substituting healthier options, planning their meals ahead [22], eating more fruits and vegetables, complying with dietary guidelines [24], and eating fewer unhealthy foods [23, 24]. Yet this positive rise in nutrition awareness is limited by confused feelings regarding the precise issues that nutrition needs to tackle during pregnancy. Pregnant women perceive a fragmentation between their “self” and “their pregnancy” which influences the control and acceptability of gestational weight gain [25]. They feel stigmatised by healthcare providers regarding their weight gain [26] and constantly need to justify their behaviour [25]. In this context, issues related to weight gain may outweigh other nutrition-related concerns. A recent qualitative study on the dietary behaviours of Swedish pregnant women showed that nutrition-related issues could be associated with negative feelings such as fear, guilt and uncertainty [11]. Finally, there is still a paucity of data on how pregnant women perceive their control of eating behaviour and how the combination of all nutrition-related issues might shape the trajectories followed by all pregnant women (not restricted to obese or overweight women) towards healthier eating behaviour during pregnancy.

The main objective of this exploratory study was to better understand the determinants of eating behaviours in French pregnant women by focusing on their concerns, attitudes and beliefs and their nutrition-related information seeking practices.

## Methods

### Study design

This paper reports on data collected during a qualitative study designed to investigate the nutrition concerns, beliefs and attitudes of French pregnant women, their nutrition-related information seeking behaviour and their need to benefit from tailored dietary advice during pregnancy. Focus groups were used to collect these data. The conduct and reporting of this study complied with the guidelines outlined in the consolidated criteria for reporting qualitative research (COREQ) [28]; all details are provided in Additional file 1.

### Recruitment of participants

We conducted seven focus group sessions that involved a total of 40 French pregnant women: five sessions in Paris (Ile-de-France, France; n = 27) and two in Aix-en-Provence (Provence Alpes Côte d’Azur, France; n = 13). The criteria for eligibility required that women should be pregnant, French-speaking, had not developed gestational diabetes and were not experiencing a multiple pregnancy. Because the objective of this study was to elicit verbal interactions on the subject of diet and nutrition between pregnant women from various familial, social and dietary backgrounds, each session involved pregnant women whose pre-pregnancy body mass index (BMI), parity and socio-occupational status all differed. The characteristics of all the participants are shown in Table 1.

**Table 1:** Characteristics of participants by region of recruitment

	Aix-en-Provence (*n* = 13)	Paris (*n* = 27)	Total (*n* = 40)
Age^a^ (years)	31.9 ± 5.5	29.7 ± 3.4	30.5 ± 4.2
Pre-pregnancy BMI^a^ (kg/m^2^)	21.8 ± 3.0	22.5 ± 3.7	22.2 ± 3.4
Trimester of pregnancy^b^			
1^st^ 2^nd^ 3^rd^	15.4 % (2) 23.1 % (3) 61.5 % (8)	11.1 % (3) 63.0 % (17) 25.9 % (7)	12.5 % (5) 50.0 % (20) 37.5 % (15)
Primiparas^b^	46.2 % (6)	51.9 % (14)	50.0 % (20)
Household income^b^ (€ per month)			
<2000 2000–4000 >4000 Don’t wish to answer	23.1 % (3) 38.5 % (5) 30.8 % (4) 7.7 % (1)	11.1 % (3) 55.6 % (15) 22.2 % (6) 11.1 % (3)	15.0 % (6) 50.0 % (20) 25.0 % (10) 10.0 % (4)
Had previously followed a diet^b^			
Never Once Several times	30.8 % (4) 30.8 % (4) 38.5 % (5)	51.9 % (14) 11.1 % (3) 37.0 % (10)	45.0 % (18) 17.5 % (7) 37.5 % (15)

^a^All values are mean ± SD

^b^All values are percentages followed by the corresponding number of participants between brackets

### Data collection

Each 120-min session was video-recorded and conducted according to standard procedures for focus groups [29]. The first and last authors (CB and PG) designed an interview guide that included the key topics to be investigated after a literature review and consultation with the project team. The guide focused on three main topics: (1) concerns, beliefs and attitudes towards diet and nutrition during pregnancy, (2) nutrition-related information seeking behaviour and (3) need to benefit from tailored dietary advice. Only data regarding topics (1) and (2) are considered in this study. A summary of the key questions of the topics (1) and (2) in the interview guide is presented in Table 2. All the questions were open-ended. The first author (CB) was the moderator who led all the sessions. An assistant moderator attended each session to assist with note taking, time management and video-recording, as well as dealing with issues such as non-verbal interactions between the participants. Participants received an incentive payment of €40 after completion of the study. Data collection was ensured by the first author between March and June 2015.

**Table 2:** Key questions in the interview guide

*Summary of key questions*
Topic 1 : Concerns, beliefs and attitudes towards diet and nutrition during pregnancy
If you heard the phrase “eating while pregnant”, what would be the first thing you would think of? Have you in practice changed in your food habits since you became pregnant? What do you think about the well-known saying "eating for two when you are pregnant"? What do you think about food restrictions during pregnancy?
Topic 2 : Nutrition-related information seeking behaviour
Where did you obtain all your nutrition-related information? Among the information sources you have quoted, which one appears to be most relevant? Among all information sources you have quoted, which one appears to be the most reliable? Why do you think it is the most reliable? On the other hand, which one appears to be the least reliable? Why do you think it is the least reliable? Do you look for nutrition-related information yourself? What type of nutrition-related information are you looking for?

### Data analysis

All focus group discussions were transcribed in full by the moderator. As no previous study had been performed on the eating behaviours of French pregnant women, we did not declare any pre-determined theory before data collection. An inductive thematic approach, adapted from the grounded theory, was therefore implemented to analyse the data. This approach involves familiarisation with the data, an open-coding process and data interpretation in themes derived from identified codes [30, 31]. The transcripts were double-coded independently by the same two researchers (CB and GLG) using Nvivo 11 Pro for Windows (QSR International Pty Ltd, Victoria, Australia). Discrepancies between the two researchers regarding the coded categories were identified through the software and resolved through discussion; the final codebook was then defined. The coded data were then grouped in two major themes and their sub-themes (Table 3). The first theme related to the eating behaviour of pregnant women was analysed using the attribution theory. Identified sub-themes were defined whether their causes were attributed to an outside event or person (external) or to themselves (internal) by the pregnant women [32]. The second theme concerned nutrition-related information seeking behaviour and was divided into three sub-themes: the two aspects of information behaviour (the passive absorption of information and active information seeking) and the selection of information to integrate in their eating behaviour [33]. Each quotation illustrating the themes and sub-themes was identified with the participant’s number followed by the quotation number, and these are presented in French in Additional file 2. Characteristics of each participant according to her number are presented in Additional file 3.

**Table 3:** Themes and subthemes identified

Themes	Sub-themes
*Eating behaviour of pregnant women*	External attribution: 1. Foods changing from being usual to dangerous - The “impressive and frustrating list of forbidden foods” - After frustration come anxiety, fear and then guilt - Norms *versus* personal practices 2. Physiological changes impacting food intakes - Pregnancy pains that restrict women’s food choices - Pregnancy-related physiological changes that modify food choices 3. Weight gain - Losing control over body weight - External surveillance of weight gain - Reassuring themselves Internal attribution: 4. The empowerment endeavour: building a healthier diet for mothers and their babies - Strategies to achieve a healthier diet - Maintaining some food indulgences - Setting personal goals to internalise weight gain - Contributing to their own and their baby’s health and well-being : the beginning of motherhood
*Nutrition-related information behaviour*	1. Passive absorption of information - From healthcare providers - From their social environment - From the mass media - Difficult cross-checking of information from all sources 2. Active information seeking behaviour - Benefiting from the experiences of people in their social environment - Ambiguous use of the Internet - The ultimate step: asking their healthcare provider for guidance 3. Translating information into eating behaviour

## Results

### Eating behaviour of pregnant women

1. External attribution: foods changing from being usual to dangerous
*The “impressive and frustrating list of forbidden foods”*
    For food safety reasons, the consumption of some foods is strongly discouraged during pregnancy. Most participants reported that the healthcare provider monitoring their pregnancy provided them with “*an impressive list of forbidden foods”*.
*“My gynaecologist gave me a list, telling me all the different food I couldn´t eat […]. She told me: “You must adhere strictly to the instructions written on this list, stick it on your fridge and check everything that goes in and out!”” (P31-1)*
    Both the lack of explanations, and the quantity and diversity of the food items usually eaten in France that were included on this list, resulted in the development of considerable frustration among the participants who wanted to follow these guidelines, because they had a major effect on their daily dietary habits.
    During social events, as well as being frustrated, some participants revealed that they felt excluded because they could not eat the same things as everyone else. In the French context, where eating is closely linked to sharing, this issue was problematic.
*“During my last pregnancy, I was quite frustrated in Christmas because I couldn´t eat smoked salmon like everyone else around me.” (P62-2)*
*After frustration come anxiety, fear and then guilt*
    The discourse of our pregnant women also highlighted their anxieties and fears regarding food consumption. The list of inadvisable items could not be exhaustive and specific. They became worried about eating meat that might not have been cooked enough, a cheese they had not identified as being potentially “forbidden”, or raw vegetables that had not been washed thoroughly enough.
*“I have a question: I know goat’s cheese is not allowed, but for example if it is cooked is it okay then?”*
*“For example, goat cheese logs that are pasteurised, no worries.” (P53/P56-3)*
    We identified three sources for this anxiety. First and foremost, they were anxious that risky eating behaviour might affect their baby’s health which would mean that they were not a “good” mother. Furthermore, the risks were unclear because they could not be well identified and evaluated. Lastly, participants felt they were sometimes being judged by their relatives regarding their food consumption. People in their social environment had heard about foods whose consumption is not recommended during pregnancy, so they questioned them, thus exacerbating their guilt.
*“I think to myself that I would like to eat that, because the main aim is to have a healthy baby anyway, […] When people say, “Ah! But why are you eating that?”, I feel guilty. […] But I am being careful; if I have any doubts, I don’t eat it.” (P22-4)*
*Norms versus personal practices*
    A great majority of our participants were aware of these norms. They might allow themselves some deviance from these strong and confusing norms. However, each deviance needed to be justified, in order to cope with the guilt it induced of not being a “good” mother. Participants mostly justified any deviances by mentioning their healthcare providers (*“My gynaecologist told me that eating sushi was all right.” (P75-5)),* their usual and general compliance with these norms, or their past experiences (*“In the end, there were no effects on many other pregnancies.” (P33-6)*), particularly in multiparas.
2. External attribution: Physiological changes impacting food intakes
  Our participants reported many physiological changes that impacted their food choices, resulting in modifications to their pre-pregnancy diet. These physiological changes led to a variety of constraints affecting their daily life, including their food choices. They were perceived as “external” because they were an inherent part of pregnancy, not due to their personal will.
*Pregnancy pains that restrict women’s food choices*
    On the one hand, when nausea, gastroesophageal reflux, loss of appetite or food aversions due to an increased sensitivity to odours were experienced by participants, they were perceived as pathological situations that were difficult to manage in their daily life and restricted their food choices. They could be referred to as “pregnancy pains”. All participants had experienced at least one of these negative physiological changes.
*“I always feel sick, due to foods, or smells, or other things like that, it´s horrible! […] There are some things that disgust me, such as my daughter’s favourite cheese; when I give her some, I could cry, it’s so disgusting!” (P31-7)*
*Pregnancy-related physiological changes that modify food choices*
    On the other hand, enhanced sensations, changes to food preferences or food cravings were perceived as non-pathological situations that formed part of their daily experience of pregnancy and caused changes to their food choices. Food cravings were very common among participants. They mainly concerned sweet products or very specific foods that differed from the women’s usual diets, and usually had to be satisfied as soon as possible.
*“I had some weird desires, such as spring rolls and vanilla dessert […] So for a while I ate a lot of spring rolls and vanilla desserts, even though I had not been a particular fan before.” (P24-8)*
    Finally, the participants considered these changes to be normal because they were pregnancy-related, so even if they did affect their food choices, they accepted them as a response of their body to pregnancy.
*“But I think whatever we do, there are pains that we’ll always have.” (P26-9)*
3. External attribution: Weight gain
  Pregnant women in our study claimed that weight gain is a “normal” process that is inevitable during pregnancy, the only time in their lives when they could put on weight. It represented a non-pathological, pregnancy-related physiological change in the same way as nausea or food cravings, except for its visible and quantifiable aspect. As a physiological change, they perceived it as “external” because it was beyond their own will.
*Losing control over body weight*
    Participants revealed that they felt they were losing control over their weight. They experienced weight variations more than usual. The expected ideal monthly weight gain was far from what the participants actually experienced. They attributed weight gain to a quantifiable but not easily predictable response of their body to pregnancy.
*“It’s difficult when you are pregnant [to manage your weight gain]: the body is in command and I really feel like I lost control…” (P16-10)*
    If they did not put enough on weight, they worried about having an undernourished child with a low birth weight. Putting on weight during pregnancy was perceived as “normal”, so that a low weight gain could be seen as an absence of the most visible and well-known characteristic of pregnant women.
*“[…] I’ve only put on one kilo since I have been pregnant! […] People around me are worried, because a pregnant woman is supposed to put on weight. […] But then the doctors reassured me. […] However, it can be disturbing, because unconsciously…” (P53-11)*
    However, if they put on weight too much, they were worried about keeping it after the birth. Multiparas who had experienced excessive gestational weight gain during previous pregnancies tended to be more stressed by weight gain than primiparas, because of the problem of going on a postpartum diet. However, neither primiparas nor multiparas wanted to deprive themselves or their child for reasons of appearance.
    “*I’m also trying to be careful, because losing weight afterwards is not at all easy. During my previous pregnancies, I gained nineteen and seventeen kilos. But that’s the way it goes, we can’t starve either!” (P62-12)*
*External surveillance of weight gain*
    In France, pregnant women benefit from a monthly visit to a healthcare provider. At each monthly appointment, they are weighed by the gynaecologist or midwife, who will judge whether their weight gain is appropriate. If their weight gain is considered to be more than expected from the previous appointment, healthcare providers clearly advise them to eat less.
*“Even if you don’t want to check it yourself, the gynaecologist will weigh you every month!” (P64-13)*
    According to the participants, this monthly check could be stressful, because of the gap between the external controllability they perceived on their weight gain and the internal controllability the healthcare provider attributed to it. From a health perspective, they knew that adequate weight gain is critical to limit adverse pregnancy outcomes, such as gestational diabetes mellitus or birth complications, and to optimise foetal growth. Nevertheless, the reaction of healthcare providers to excessive weight gain could be very guilt-inducing and traumatic for the women.
*“[For my first pregnancy] on the day of birth, I was scolded, and the doctor called me a big fat cow.” (P25-14)*
*Reassuring themselves*
    Most of the pregnant women tried to reassure themselves and justify their weight gain by following the traditional belief that whatever their weight, and contrary to what they had been told, there was no clear relationship between maternal weight gain and the baby’s weight at birth. According to the participants, the foetus drew on its mother’s reserves to satisfy its needs.
*“But the baby’s weight isn’t linked to your weight gain.”*
*“No, the weight we gain doesn’t mean anything.” (P41/P42-15)*
4. Internal attribution: The empowerment endeavour: building a healthier diet for mothers and their babies
*Strategies to achieve a healthier diet*
    All participants stated that they were more aware of nutrition during pregnancy, and identified pregnancy as a period during which they should *“eat more healthily than before” (P15-16)*. Most participants said they had an increased appetite and they had been eating more since the start of their pregnancy. However, although they agreed they were eating more than usual, above all they wanted to eat better and not to excess. “*Eating for two*” was far from the participants’ perceptions of their pregnancy, which they tended to attribute to old misconceptions, and this had turned into “*Eating for two in quality but not in quantity”*.
    Three categories could be identified regarding the adoption of healthier eating behaviour: the nutrient adequacy of the diet, the dietary planning and the origins of their food (Table 4).

**Table 4:** Classification of strategies to achieve a healthier diet during pregnancy

Strategies	Categories		
	Food origin	Nutrient adequacy of the diet	Dietary planning
Eating more fruits and vegetables: *“I force myself to always include fruits and vegetables in my diet, at breakfast, lunch and dinner.” (P31-17)*		X	
Balancing the diet: *“I really didn’t care before! Now I am trying to eat more fish, more vegetables.” (P55-18)*		X	
Favouring foods known to be rich in specific vitamins and minerals: *“I have added things to my diet that I did not eat much, such as lentils to obtain iron.” (P33-19)*		X	
Substituting unhealthy foods with healthier options: *“Even if I continue to snack, I’ll eat a dairy product, something more balanced, more sensible.” (P42-20)*		X	
Eating less sugar: *“I have really backed off sugars because I was too addicted before.” (P13-21)*		X	
Eating three or four meals a day: *“Before being pregnant, I was on one or even two meals a day and now I try to do three regular.” (P24-22)*			X
Favouring organic, local or farm foods: *“As for meat, I always chose the best quality cuts from the butcher’s anyway […] and the same goes for vegetables. Now I’m more into buying directly from the producer […] and finding more organic foods.” (P11-23)*	X		
Splitting food intakes: *“I skip dessert at lunch-time, and then eat it at around three or four pm.” (P33-24)*		X	X
Avoiding processed products, above all ready-to-eat meals: *“I buy fewer processed foods, or only the least processed items.” (P67-25)*	X	X	
Cooking more: *“We also try to do more ‘home-made’ dishes, because then I know what has gone into them.” (P41-26)*	X	X	X
    The degree of application of these strategies varied among participants. Even those who said they were not very interested in diet and nutrition made some changes to their usual diets. While primiparas might report an unhealthy pre-pregnancy diet, most multiparas explained that the food habits of their first pregnancies had been partly maintained. Dietary supplements were not favoured in order to consume more vitamins and minerals; they gave priority to foods rich in vitamins and minerals.
*Maintaining some food indulgences*
    Food indulgence was critical to participants’ well-being as they felt sufficiently restricted already. They allowed themselves to eat some treats, such as sweet or chocolate products, because “*chocolate is the only thing [they] can eat that [they] know is safe” (P12-27)*. They felt that being too frustrated would impact their well-being, which they associated with that of their baby.
*Setting personal goals to internalise weight gain*
    They felt powerless regarding weight gain, but they nevertheless set personal goals in order to limit it. So pregnancy meant not only “*weight gain*” but “*weight gain management*”. Eating a healthier diet allowed them to attain these goals even if their appetite increased. This justified their weight gain and reduced their guilt.
    “*The cookies I dream about I never buy it not to break down, not to grow fat.” (P31-28)*
*Contributing to their own and their baby’s health and well-being: the beginning of motherhood*
    The ultimate aim of a pregnant woman is to give birth to a healthy baby. Adopting a healthier diet is one of the first actions they can take to achieve this. Our participants naturally wanted the best for their babies and they felt responsible for what they ate because they knew that *“everything [they] eat the baby will eat with [them]” (P41-29).* They sometimes allowed themselves to eat unhealthy foods, but then they felt guilty about feeding their baby in an unhealthy manner.
    “*For example, when I eat a fast food meal […], I tell myself that it’s not nice what I am doing to my baby […] It’s crammed with additives, it's not even real food!” (P56-30)*
    Adopting healthier eating behaviour can positively impact the baby’s health and well-being, and represents a first step into motherhood. Pregnant women start to act like a mother and take responsibility by controlling the baby’s diet.
*“But there are two of us now, you become less egocentric! […]. Because I can’t control anything because she is growing alone, let’s say that [diet] is the only thing I can manage. I can’t interfere with the colour of her eyes or hair, but I share responsibility for her well-being.” (P52-31)*
    Their own health was also an objective, because if an inadequate intake of nutrients could harm the baby, it could also lead to complications during delivery and afterwards. The self-fulfilment of our participants, and their well-being as women were crucial to their perception of being a “good mother”.
*“It’s also important after the pregnancy, because you’re a mum, you’re a woman, you’re a wife, you’re professional and for me that’s something that’s super important. To be a fulfilled mum, I need to be a fulfilled woman; I think it starts from your diet, so I need to feel good in my body if I am to be fulfilled as a mum.” (P74-32)*

### Nutrition-related information seeking behaviour

In the next section, we focus on the nutrition-related information that the women absorbed passively or sought actively, and specifically during pregnancy. Nutrition-related information sources can be divided into three categories: (1) healthcare providers, (2) the social environment and (3) the mass media.

1. Passive absorption of information
*From healthcare providers*
    According to pregnant women involved in our study, their healthcare providers were mainly the gynaecologist and/or the midwife. The participants identified nutrition as a component of their babies’ health and well-being, so they strongly trusted their healthcare providers who appeared to be the gold standard. However, professionals did not spend much time discussing nutrition-related issues. In most cases, they quickly referred to dietary restrictions during pregnancy, being focused mainly on the patient’s weight gain. In the event of excessive weight gain, they might refer pregnant women to a dietician. Pregnant women benefited from very little information on the foods they should favour, and the advice was much more about negatives than positives.
*“They don’t all remember to tell us if we are suffering from a deficiency, or suggest that we should eat this or that!” (P52-33)*
    During their antenatal care, pregnant women often saw different healthcare providers, whose interest in and opinion on nutrition-related issues could vary. Conflicting information on nutrition-related issues led to incomprehension and irritation, and thus increased their anxiety.
*“Among doctors, there are two sorts of stories: some say yes, others no. […] my gynaecologist is against dietary supplements, but when I arrived at the maternity hospital, [the midwife] gave me a prescription [to take dietary supplements]. What am I expected to do?” (P53-34)*
*From their social environment*
    In their social environment, relatives very often advised participants about nutrition, based on their experience or on hearsay. Because “*everyone has their own story*” *(P14-35)* about nutrition-related issues (without any scientific background), trying to cross-check this information also resulted in inconsistency.
*“Friends and relatives talk about their own stories and they draw hasty conclusions. One person told me I should eat a yoghurt every day, otherwise […] my child would not be able to deal with the sun!” (P32-36)*
    The social environment was not perceived as a reliable source of information, except for their mothers or those of others. Pregnant women were more receptive to the transmission of health or cooking habits from their mothers.
*“I have learned from my mother what to do and how to cook.” (P15-37)*
*From the mass media*
    Pregnant women are becoming more receptive to general information on food and nutrition gained from the mass media. More and more reports are broadcast on TV or published in the press on both the nutrition and safety aspects of the diet, and they often highlight the unhealthy aspects of products and processes used by food industries. Such information can create anxiety with respect to food. Although this information was not pregnancy-specific, participants felt more receptive towards it.
*“I stopped watching TV shows because I began to feel that I couldn’t eat anything at all! […] Each time they come up with a new problem!” (P11-38)*
*Difficult cross-checking of information from all sources*
    Participants tried to deal with the information on nutrition-related issues they obtained from all these sources, to which they gave more or less credence. The inconsistencies they perceived within a source was heightened when they cross-checked information between different sources.
*“Health professionals tell us specifically not to eat for two and to eat as usual. But our families and friends tell us to have second helpings because there are two of us. I experienced this again at the week-end.” (P52-39)*
    In reaction to this confusion, most of them developed an information seeking behaviour so that they could achieve nutrition-related empowerment.
2. Active information seeking behaviour
  We identified two types of nutrition-related information seeking behaviour: searching at any time to obtain general nutritional knowledge and searching specific information in response to a pregnancy-related problem or question. Multiparas tended to seek less general information, relying on their previous pregnancy experience. However, because “*every pregnancy is different” (P42-40)*, they felt they had not benefited from or sought enough nutrition-related information during their previous pregnancy.
*Benefiting from the experiences of people in their social environment*
    Nutrition-related information seeking behaviour was implemented during interpersonal exchanges with other mothers (especially their own mothers) or pregnant women in their social environment. However, their mother’s experiences were judged as being in the past, and the updating of information was questioned.
*“I asked [my mother] lots of questions at the start. But each time, she told me that it had been a long ago for her […] and at that time, it was much easier, pregnant women could eat whatever they wanted!” (P23-41)*
*Ambiguous use of the Internet*
    Nutrition-related seeking behaviour mostly concerned the mass media and particularly the Internet, but use of the latter tended to be ambiguous. On the one hand, it was the first idea in the participants’ minds because of its immediacy, continuous availability and the assurance of finding at least one answer to their question.
*“On the web, you can find information straight away, when you want results they are immediate, you don’t need to wait for the next appointment with the midwife.” (P42-42)*
    However, they did not really trust this easily accessible information because of the lack of identification of its sources and the countless and conflicting numbers of articles and forum discussions. Often, instead of answering to their questions, this heightened their confusion. However, they drew a distinction between generic and professional websites, which were considered to be trustworthy, because the authors could be identified.
*“On the web, apart from really specialised websites, there are some sites when you don’t know what to believe.” (P42-43)*
*The ultimate step: asking their healthcare provider for guidance*
    When a specific health problem occurred, an internet search was considered as a first step, but the findings had to be confirmed by a healthcare provider.
*“[…] it is necessary to check the information, either with a specialist or with someone else. The web cannot be the only source of information…” (P12-44)*
    Aside from health problems, they felt it was not appropriate to bother their midwives or gynaecologists with questions about nutrition.
3. Translating information into eating behaviour
  This active search for information increased the confusion experienced by our participants. They were not satisfied with the information they received and sought, and needed to reaffirm their empowerment regarding their eating behaviour. Participants therefore used their common sense in order to filter information depending on the perceived reliability of both content and source, before applying it to their diet.
*“You can talk about all the sources of information on diet during pregnancy […] but in the end, it is up to you to make the right decisions!” (P53-45)*
  Furthermore, pregnant women in our study already had a basic knowledge of the components of a healthy diet. They were able to remobilise nutrition knowledge they had absorbed passively before pregnancy, and translate it into their eating behaviour.
*“You already know it instinctively, you know that you should not eat too many fatty or sweet foods; you should be eating vegetables. We are aware of the basic ideas [of a healthy diet].” (P12-46)*

## Discussion

**Fig. 1:**
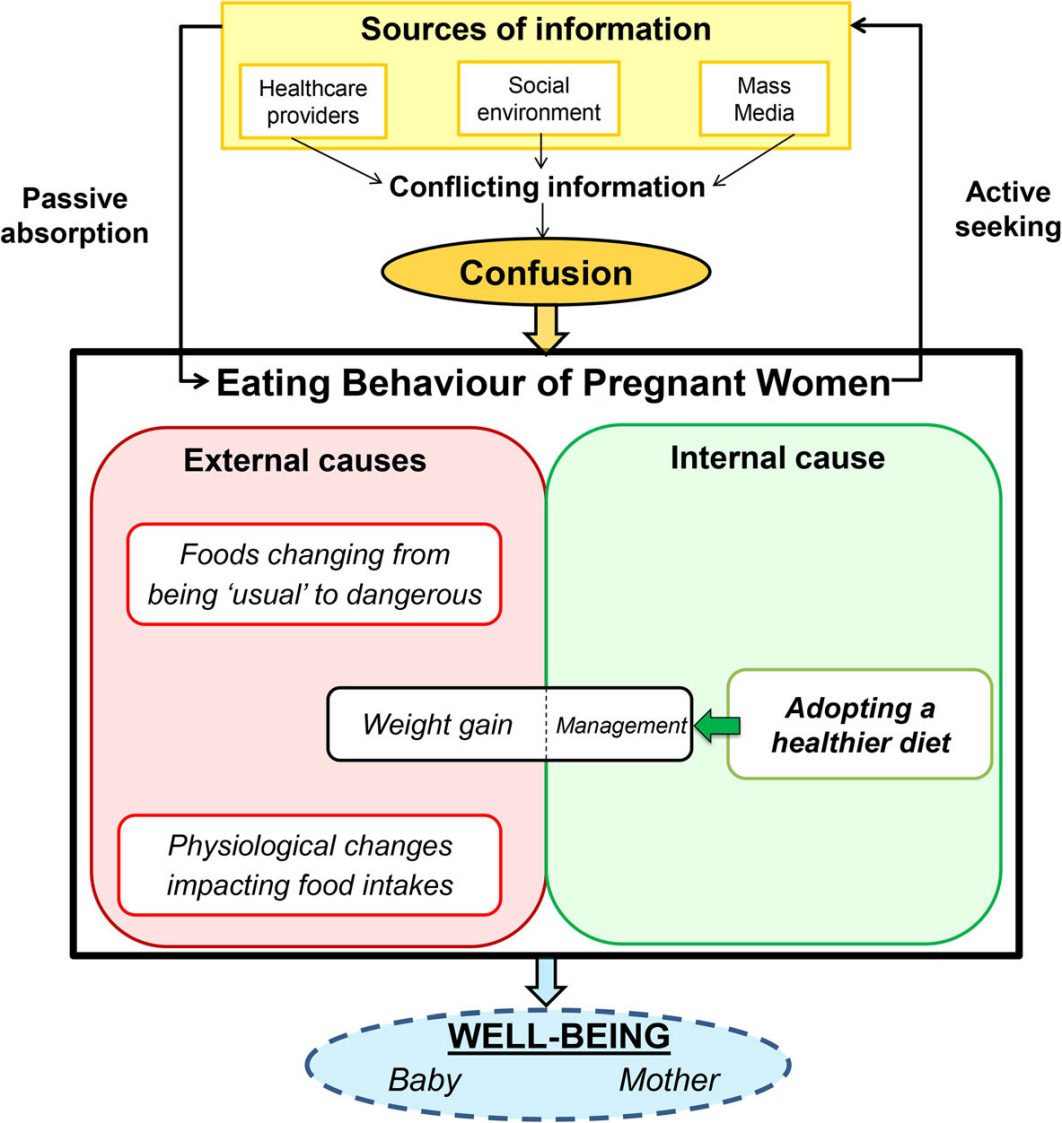
Diagram of the eating behaviour and the nutrition-related information seeking practices of pregnant women participating in our study (n = 40)

During this study, we were able to demonstrate a rise in nutrition awareness among French women during pregnancy, in line with what has been reported in other developed countries [4–6, 11, 24]. The novel finding of this study was the description of the entire process resulting in the adoption of a healthier diet by French pregnant women, highlighting the importance of their empowerment with respect to diet and nutrition (Fig. 1). These women saw adopting a healthier diet as a first step into motherhood, a means of starting to care for their baby before birth, and also as a way of regaining control over their diet and their body. Indeed, many elements that were out of their control restricted their food choices. This study also emphasised the fact that French pregnant women received heterogeneous and conflicting information on nutrition-related issues, which led them developing active information seeking behaviour. However, the perceived confusion between and within information sources jeopardised the adoption of a healthier diet for them and their baby.

In this population, nutrition awareness cannot be reduced to simply gaining knowledge about nutrition-related issues. It becomes “hot” or “active”, i.e. their latent cognition becomes more salient, they become more preoccupied by nutrition as a subject of “continuous attention” and “deliberate supervision” [5]. Pregnant women translate their knowledge into behaviour and then make dietary modifications. These modifications may be more or less intense, depending on individual lifestyle trajectories. Indeed, there are women who will “continue the same way” [6], but in our study they represented a minority. As in other qualitative studies including normal-weight women, most of our participants could be characterised as “going all the way” or “taking the flexible way” [6] and experienced major shifts in their nutrition awareness. A quantitative study performed in a small group of French pregnant women had concluded to an increase in the consumption of fruits and vegetables and a reduction in sugar consumption when compared with the prenatal period [34], thus supporting our qualitative findings in French women. Moreover, even if all these modifications were not sustained during the postpartum period by multiparas, changes to eating behaviour during a first pregnancy were partly extended and transmitted to other members of the family. Pregnancy thus offers a window of opportunity to adopt healthier eating behaviours, which argues in favour of the life course perspective [1].

However, this increase in nutrition awareness does not simply relate to healthy eating. This period of life is marked by nutrition-related tensions, the causal attribution of which is external from pregnant women. Because of pregnancy-related physiological changes [35, 36] and food restrictions [37, 38] women need to reconsider their diet in early pregnancy. Although pregnancy-related physiological changes are out of a woman’s control, they are well accepted because they are perceived as a typical feature of pregnancy [35]. By contrast, the norms concerning food restrictions are numerous [39] and perceived as intrusive and oppressive. In France, where food has a strong cultural meaning, these restrictions concern foods that are very frequently consumed, such as cold cuts and cheeses [40], which can result in social exclusion and a negation of food preferences. There is considerable confusion with respect to these restrictions, which necessarily generates fear, as also demonstrated in a recent qualitative study performed in Swedish first-time pregnant women [11]. They do not know precisely which specific foods they cannot eat [11, 39] but they know that eating something “wrong” might be harmful to their babies [11]. More than just dietary norms, “forbidden” foods are a component of the nutrition-related “mothering norms” that were identified by Copelton et al. [41]. The sacrifice of food preferences represents one of the starting points of motherhood, and pregnant women will prefer to deprive themselves for a while rather than risking any harm to their baby and acting as a “bad” mother [41]. Nevertheless, most pregnant women allow themselves some transgressions in exceptional cases, but it appears they need to justify such deviances from a norm they know to cope with the guilt they will cause. Justification is intense and necessary for pregnant women in order to legitimise their eating behaviours when their deviance has questioned the ideal gestational environment they have built for their baby [25, 41].

Weight gain, like other pregnancy-related physiological changes, is inevitable during pregnancy [26]. Indeed, pregnancy could be perceived as a time to relax the rules related to weight gain that existed prior to pregnancy [25], but the external surveillance of weight gain by healthcare providers places pregnant women under pressure. Pregnant women feel judged on an issue they are finding difficult to handle and that should be “normal” during pregnancy. This also results in a justification process that refers either to biology (i.e. putting on weight is an uncontrollable bodily response to pregnancy) or to the denial of injury (i.e. weight gain is not associated with their baby’s health) [41]. Finally, the eating behaviour of pregnant women can be marked by tensions and gradually slips out of control.

Pregnant women regain control over their eating behaviour by adopting strategies to achieve a healthier diet. Many other studies have reported these dietary modifications in pregnancy, such as increasing the consumption of fruits and vegetables [24, 41, 42], complying with dietary guidelines [24], reducing the consumption of unhealthy foods [23, 24, 41], introducing healthier options, or planning their meals ahead [22]. In all studies, the health of both the baby and mother were reported as being the main objectives when adopting a healthier diet. Our findings revealed that an additional dimension, quickly mentioned in other studies [4, 6] but not deeply investigated, was operating in the behaviour of these women: the well-being. Introduced by Block et al., the “food well-being” paradigm, defined as a “positive psychological, physical, emotional and social relationship with food at both the individual and societal levels”, provides a new dimension when considering food. While “food as health” is “paternalistic and normative” and made up of “restraints and restrictions”, “food as well-being” is “holistic and integrative”, “consumer oriented” and based on a “positive approach” [43]. Acting positively as a “good” mother of their own will, constitutes a re-appropriation of eating behaviour apart from external norms or uncontrollable situations. In this empowerment process, their behaviour is mainly baby-focused but it is also self-focused, because this makes weight gain management possible and acts positively on their well-being. Pregnant women want to follow a diet “which is good for their child or them as a mother” and “which is good for them as a woman” [44]. Fragmentation of the self between “me” and “my pregnancy” [25] or between “me as a woman” and “me as a mother” [44] seems to be the foundation for constructing an identity during pregnancy. Our findings showed that a reunification could exist through the adoption of a healthier eating behaviour, not the healthiest behaviour. Gradually, the guiltless idea is emerging that self-fulfilment and well-being as a woman is crucial to being first a “good expectant mother” and then a “good” mother.

It should be noted that the pregnant women in our study already knew a lot about general nutritional principles. Before their pregnancy, these women had heard public health messages about nutrition [45] but these were only applied to their diet in a limited manner. If women passively absorb this information before their pregnancy, all nutrition-related concerns that this physiological, social and emotional transition may trigger will place them in a more “active” position [6, 8]. Developing knowledge about nutrition should result in empowerment, but from the three types of information source identified during pregnancy (healthcare providers, the social environment and the media) [8], they found little reliable and pregnancy-focused information on nutrition-related issues. The gold standard represented by healthcare providers was somewhat tarnished when it came to questions on nutrition-related issues. Healthcare providers tend to focus on “hard” health issues, with easily measurable effects and observed within a short period. Thus, restriction and surveillance tend to be stronger than support [46]. They lack the time, resources and training to advise pregnant women about nutrition [10, 26]. Consequently, nutrition-related information is scarce and may vary from one healthcare provider to another. Finally, pregnant women feel they should not bother them by asking for guidance on issues not related to “hard” health problems or laboratory deviances [11]. Healthcare providers remain tied to “food as health”. Disappointed by the advice they did or did not receive from healthcare providers on nutrition-related issues, pregnant women might seek for information by asking for guidance from their mothers or other veteran mothers in their social environment [8, 11, 42, 47], but this raised questions as to whether this information was up to date and thus reliable. Finally, active nutrition-related seeking behaviour was mainly implemented via the Internet, because of its ease of access, continuous availability and immediacy. Most of pregnant women in our study had searched at least once for nutrition-related information on the Internet, which, paradoxically was the most widely used source but not the most reliable, as was also found by other studies [8, 9, 11, 42]. Indeed, the quantity of information available via the Internet is considerable, but also conflicting and coming from unidentified sources [26, 48]. The nutrition-related information received and sought by pregnant women was mainly restrictive, frustrating, and above all contradictory within and between sources. This could lead to confusion, thus complicating the adoption of a healthier diet. Pregnant women would expect support for mitigating nutrition-related tensions.

### Strengths and limitations

Our use of a holistic diet perspective to investigate the eating behaviour of pregnant women was the major strength of this study. By focusing not only on weight gain it was possible for us to describe eating behaviour during pregnancy as a whole. The weight gain perspective had previously been chosen for many qualitative studies designed to investigate nutrition-related issues during pregnancy, especially in the UK and US, where they were mainly performed in overweight or obese women [22, 23, 27, 42, 49]. In our sample, 75 % of the women had a normal weight before their pregnancy, similar to that reported among French women aged between 18 and 44 years (63–82 %) [50], so that other components of diet during pregnancy appeared to be of great importance for these women. In our study, the attribution theory [32] was useful to understand how nutrition-related issues are perceived by pregnant women and to reveal the problems they encounter in internalising them.

Regarding the method, our qualitative approach enabled us to collect deeper information rather than produce a quantitative survey. This was particularly relevant in our groups, where the women shared a major, visible and also very personal feature: their pregnancy. Furthermore, groups were organised in two different regions of France: in the countryside in the South and in Paris, which allowed us to gain access to populations with different ways of life and eating habits.

The focus group method chosen for this study could have been problematic. Our focus groups involved women at different stages of their pregnancy and of different parity. This choice was deliberate because it favoured interactions and the transmission of information between participants. However, groups that were homogeneous with respect to stages of pregnancy and parity could have been useful to investigate differences in nutrition awareness and perceptions of nutrition-related issues across trimesters of pregnancy or between primiparas and multiparas. Another limitation of this study may have been that the women who were recruited were informed about the main topic of discussions. This initial knowledge may have induced a recruitment bias, because the women who volunteered to take part in the discussion might have been more interested in nutrition-related issues than other pregnant women. However, the incentive we offered may have enabled a reduction in this bias.

## Conclusions

In the life course perspective, pregnancy is a transition period that offers a window of opportunity for women to change towards healthier diets which can result in improving the health and well-being of both the mother and baby. Although we observed a rise in nutrition awareness during pregnancy among French women, we found that diet during pregnancy is restricted by elements that are out of the control of pregnant women. Women had received a great deal of information on nutrition during pregnancy, but had also developed active nutrition-related information seeking behaviour as a way of regaining control over their diet and building a healthier diet for them and their baby. However, the confusion they perceived clearly limited the adoption of a healthier diet.

### Implications for practice

Positive dietary information is critical to dietary self-negotiation and empowerment. In France, dietary guidelines relative to antenatal care, that could be used as a basis for antenatal counselling by the gynaecologist or the midwife responsible for the follow-up of the pregnancy, are mostly based on food restrictions and weight gain surveillance [51]. It is necessary to take account of nutrition-related tensions during pregnancy and to help pregnant women to relieve these by developing positive communication on a healthy diet, focused on the well-being of both the baby and mother. This could lead to the adoption and maintenance of a healthier diet because it will be positively internalised. Our pregnant women identified their healthcare providers as the most reliable source of information on nutrition-related issues, so these professionals could be the best vehicle to disseminate positive information on nutrition, without neglecting surveillance and monitoring. As gynaecologists and midwives do not have a specific nutrition background, they could collaborate with dieticians and advised pregnant women to meet with a dietician during individual or group sessions to receive nutrition counselling adapted to their pregnancy.

### Implications for future research

Dietary modifications during pregnancy are not well characterised. A longitudinal study would be useful to evaluate food intakes of women during the specific period of their lives between preconception and birth. As reported in our study, multiparas generally seemed to have a healthier preconceptional diet than primiparas, so a comparison at this specific time between multiparas and primiparas would also be necessary to provide elements in favour of the life course perspective.

Furthermore, a randomised controlled trial would be useful to determine whether the provision of positive information (i.e. tailored dietary counselling provided by a dietician or by an adapted mHealth or eHealth program) on diet during pregnancy would impact the eating behaviour of pregnant women and result in mitigating nutrition-related tensions and improving nutrient intakes of pregnant women.

## Additional files

Additional file 1: Details of the study relative to the research team and reflexivity, study design and analysis and findings according to the consolidated criteria for reporting qualitative research (COREQ) [28]. (DOCX 16 kb)
Additional file 2: Quotations in French (native language of participants) identified with the participants number followed by the number of the quotation as presented in the article. (DOCX 20 kb)
Additional file 3: Identification number, session, recruitment place, trimester of pregnancy and parity of each participant. (DOCX 15 kb)

## Abbreviations

BMI: Body mass index
UK: United Kingdom
US: United States of America
