# Supplementary material for: Concerns, attitudes, beliefs and information seeking practices with respect to nutrition-related issues: a qualitative study in French pregnant women
Source: BMC Pregnancy Childbirth. 2016 Oct 12;16:306. doi: 10.1186/s12884-016-1078-6 (PMC5059968; doi:10.1186/s12884-016-1078-6)
Supplement: Additional file 1: — Details of the study relative to the research team and reflexivity, study design and analysis and findings according to the consolidated criteria for reporting qualitative research (COREQ) [28]. (DOCX 16 kb) [file 12884_2016_1078_MOESM1_ESM.docx]

**Additional file 1.** Details of the study relative to the research team and reflexivity, study design and analysis and findings according to the consolidated criteria for reporting qualitative research (COREQ) [28].

**Domain 1: Research team and reflexivity**

*Personal characteristics*

The first author (CB), a PhD student whose research interest is nutrition during pregnancy, was the moderator. She was chosen as she was a young woman, but not a healthcare professional, to facilitate discussions by the participants and to limit any judgemental feelings. She had previously been trained in leading focus group interviews.

*Relationship with participants*

The moderator did not establish a relationship with the participants prior to the study. However, she had a brief telephone interview with each participant at the time of recruitment in order to describe the content of focus group sessions and briefly present herself (credentials, occupation, research interests and reasons for performing the research).

**Domain 2: Study design**

*Theoretical framework*

As no previous study had been performed on the eating behaviours of French pregnant women, we did not state a pre-determined theory before data collection. An inductive thematic approach, adapted from the grounded theory, was therefore implemented to analyse the data. This approach involves familiarisation with the data, an open-coding process, and data interpretation in the themes derived from identified codes [30, 31].

*Participant selection*

In Paris, pregnant women were recruited from consumers registered in the database of a generalist market research company. The company contacted women living in the Paris region by email, and if they were pregnant they were invited to take part in a focus group session on diet and nutrition. Those interested in participating registered with the company using an online form. In Aix-en-Provence, pregnant women were recruited using posters displayed in the gynaecologists’ waiting room in a maternity clinic (L’Etoile Maternity Clinic, Aix-en-Provence, Provence Alpes Côte d’Azur, France). They could register either by email or by calling the first author. After registering, the women were contacted by telephone by the first author in order to verify their eligibility regarding the inclusion criteria and to arrange their attendance at one of the scheduled sessions. Before each session, the first author described the context, objectives and content of the focus group session, giving information on the time, date, duration and location of the session and answering any questions about the study. In order to anticipate the drop-outs that can affect attendance at focus groups, even after registration, at least eight participants were scheduled for each session. Finally, a total of 61 pregnant women were scheduled, 21 dropped out that resulted in a total of 40 pregnant women participated in the study.

*Setting*

Each focus group session was organised in a special meeting room at the maternity clinic in Aix-en-Provence or at AgroParisTech School of Life Sciences in Paris. Only the moderator, assistant moderator and participants were present during a session. Healthy snacks and drinks were offered to the participants.

*Data collection*

Details relative to the interview guide, number of interviews, visual recordings, field notes and durations are given in the ‘Methods’ section of the article.

Following each focus group session, the moderator and assistant moderator debriefed the session together, identified the main themes to be used for the coding process and wrote a report. Three focus groups were initially carried out and transcribed. A preliminary analysis was made by the first and last authors regarding the reports on the first three first sessions, so as to identify whether thematic saturation had been reached or if the interview guide needed to be adapted because of emerging themes. They agreed to perform four more focus group sessions without modifying the interview guide. A further analysis was performed by the first and last authors, which covered the reports on all seven sessions. The first and last authors agreed that the saturation had been reached, inasmuch as no new themes had been identified by this point. A short summary of the findings was sent by email to all the participants.

**Domain 3: Analysis and findings**

*Data analysis*

Details relative to the number of data coders, the description and derivation of themes and sub-themes and the software used are described in the ‘Methods’ section of the article. After completion of the analysis, a short summary of the findings was sent to the participants by email.

*Reporting*

The themes and sub-themes are presented in the ‘Results’ section of the article. Quotations from the participants were used to illustrate the themes. The characteristics of each participant using her identification number are shown in Additional file 3.
