# Supplementary material for: Concerns, attitudes, beliefs and information seeking practices with respect to nutrition-related issues: a qualitative study in French pregnant women
Source: BMC Pregnancy Childbirth. 2016 Oct 12;16:306. doi: 10.1186/s12884-016-1078-6 (PMC5059968; doi:10.1186/s12884-016-1078-6)
Supplement: Additional file 2: — Quotations in French (native language of participants) identified with the participants number followed by the number of the quotation as presented in the article. (DOCX 20 kb) [file 12884_2016_1078_MOESM2_ESM.docx]

**Additional file 2.** Quotations in French (native language of participants) identified with the participants number followed by the number of the quotation as presented in the article.

*« […] ma gynécologue m’a balancé une liste en me disant voilà tout ce que vous n’avez pas le droit de manger […]. Elle m’a dit : « Vous vous y tenez bien, vous la mettez sur votre réfrigérateur et vous contrôlez tout ce qui rentre et tout ce qui sort ! » » (P31-1)*

*« Moi j’étais frustrée à Noël, pour ma précédente grossesse, je ne pouvais pas manger de saumon fumé et je voyais tout le monde en manger. » (P62-2)*

*« J’ai une question, je sais que le fromage de chèvre on n’a pas le droit, mais par exemple s’il est cuit est-ce qu’on a le droit ? »*

*« Par exemple, les bûches de chèvre qui sont pasteurisées tout ça, il n’y a pas de souci. » (P53/P56-3)*

*« On se dit qu’on a envie de ça mais on ne peut pas car l’enjeu c’est un bébé en bonne santé quand même. […] Quand l’entourage nous dit : « Ah ! Mais pourquoi tu manges ça ? ». Ca nous culpabilise. [...] Je suis prudente ! Je ne mange pas si j’ai un doute. » (P22-4)*

*« Mon gynécologue m’a dit que manger des sushis, ce n’est pas grave. » (P75-5)*

*« Au final, il n’y a pas eu de répercussions sur moult autres grossesses. » (P33-6)*

*« Moi j’ai toujours des nausées, par des aliments en fait, des odeurs, des choses comme ça, c’est horrible ! […] Il y a des choses qui me dégoûtent comme le fromage préféré de ma fille, quand je lui en donne je pourrais en pleurer, tellement ça me dégoute ! » (P31-7)*

*« Moi j’avais des envies un peu particulières genre des nems et de la crème dessert à la vanille […] donc pendant quelque temps j’ai mangé beaucoup de nems et de crèmes desserts à la vanille, pourtant ce ne sont pas des choses que je mangeais particulièrement avant. » (P24-8)*

*« Mais je pense que quoiqu’on fasse il y a des maux qu’on aura tout le temps. » (P26-9)*

*« Mais c’est difficile quand on est enceinte [de gérer sa prise de poids], c’est le corps qui contrôle j’ai vraiment l’impression d’avoir lâché le contrôle… »* *(P16-10)*

*« […] depuis que je suis enceinte, je n’ai pris qu’un kilo ! [...] Les gens autour de moi, ça les inquiète, parce qu’une femme enceinte est censée grossir. […]Après les médecins ils m’ont rassurée. […] Mais ça peut perturber un peu parce que dans l’inconscient… » (P53-11)*

« *J’essaie aussi de faire attention, parce que les kilos après il faut les perdre et pour mes précédentes grossesses j’ai pris dix-neuf et dix-sept kilos.* *Après c’est comme ça, on ne va pas se priver de manger non plus ! » (P62-12)*

*« Même si on ne veut pas le surveiller soi-même, tous les mois le gynécologue nous pèse ! » (P64-13)*

*« [Pour ma première grossesse] le jour de l’accouchement, je me suis faite grondée je me suis faite traitée de grosse vache par un docteur. » (P25-14)*

*« Mais le poids du bébé n’a pas de rapport avec la prise de poids. »*

*« Non, le poids qu’on prend ça ne veut rien dire. » (P41/P42-15)*

*« manger plus sain qu’avant » (P15-16)*

*« Moi je me force à avoir toujours des fruits et des légumes dans mon alimentation, le matin, le midi, le soir. » (P31-17)*

*« Je ne faisais vraiment pas attention avant ! Là je fais quand même plus attention à prendre du poisson, plus de légumes. » (P55-18)*

*« Je rajoute dans mon alimentation des choses que je ne mangeais pas trop avant pour avoir du fer, donc des lentilles. » (P33-19)*

*« Même si je vais grignoter, je vais prendre un produit laitier, quelque chose de plus équilibré, de plus raisonné. » (P42-20)*

*« J’ai vraiment levé le pied sur les sucres rapides parce que j’étais vraiment trop dedans. » (P13-21)*

*« Avant d’être enceinte, je faisais un voire deux repas par jour et maintenant j’essaie d’en faire trois réguliers. » (P24-22)*

*« Au niveau de la viande je choisissais quand même des bons morceaux chez le boucher, […] pour les légumes pareil, c’était plus l’achat au producteur directement, […] j’achetais plus de bio. » (P11-23)*

*« Je supprime le dessert à midi, et je le mange vers trois ou quatre heures. » (P33-24)*

*« Moi j’achète des aliments moins transformés, ou le moins transformés possible. » (P67-25)*

*« On essaie aussi de faire plus de « fait-maison », parce que je sais déjà ce que j’ai mis dans les aliments. » (P41-26)*

« *Le chocolat, c’est la seule chose qu’on peut manger qui ne craint pas! » (P12-27)*

« *Le cookie dont je rêve, je ne me l’achète jamais pour ne pas craquer, pour ne pas grossir. » (P31-28)*

*« Tout ce qu’on mange, le bébé le mange avec nous. » (P41-29)*

« *Par exemple quand je mange dans ne chaîne de restauration rapide, […], je me dis que c’est pas sympa ce que je lui fais à mon bébé […] c’est bourré d’additifs, c’est même pas un aliment ! » (P56-30)*

*« Mais on est vraiment deux, on est moins égocentrique ! […] comme je ne contrôle rien parce qu’elle se développe toute seule, disons que [l’alimentation] est la seule chose que je peux gérer. Je ne peux pas intervenir sur la couleur de ses yeux, de ses cheveux, mais sur son bien-être j’ai une part de responsabilité. » (P52-31)*

*« C’est important pour l’après-grossesse, parce que t’es une maman, t’es une femme, t’es une épouse, t’es professionnelle et pour moi c’est quelque chose qui est hyper important. Pour être une maman épanouie, il faut que je sois une femme épanouie et donc je pense que ça part de l’alimentation et donc j’ai besoin de me sentir bien dans mon corps pour être épanouie en tant que maman. » (P74-32)*

*« Ils ne pensent pas tous à nous signaler quand on est carencé, ils ne pensent pas à nous dire s’il faut favoriser ça ou ça ! » (P52-33)*

*« Parmi les médecins, il y a deux sons de cloches : il y en a qui disent oui, d’autres non. [...] mon gynécologue il est contre les compléments alimentaires et quand je suis arrivée à la maternité [la sage-femme] m’a fait une ordonnance [pour prendre des compléments alimentaires]. Je fais quoi alors ? » (P53-34)*

« *Chacun dit la sienne* » *(P14-35)*

*« Les amis et la famille se basent sur leur propre histoire et ils tirent des conclusions hâtives. On m’a dit qu’il fallait que je mange un yaourt tous les jours, sinon […] mon enfant il n’allait pas résister au soleil ! » (P32-36)*

*« Avec ma mère il y a une transmission dans la manière de faire et de cuisiner. » (P15-37)*

*« Moi j’ai arrêté de regarder toutes ces émissions parce que j’ai l’impression qu’à la fin on ne peut plus rien manger ! [...] A chaque fois, on nous sort un nouveau truc ! » (P11-38)*

*« Les professionnels de santé nous disent de ne surtout pas manger pour deux et de manger comme d’habitude. Et la famille, les amis nous disent de nous resservir parce qu’on est deux. J’ai encore expérimenté ça ce week-end. » (P52-39)*

« *chaque grossesse est différente »* *(P42-40)*

*« Je posais beaucoup de questions [à ma mère] au début. Mais à chaque fois elle me disait que pour elle ça faisait longtemps. […] et qu’à l’époque c’était tellement plus facile, elles mangeaient tout ce qu’elles voulaient ! » (P23-41)*

*« Sur internet l’information elle est immédiate, quand on en a besoin on a le résultat tout de suite on n’a pas à attendre le prochain rendez-vous chez la sage-femme. » (P42-42)*

*« Après sur internet, on a des sites où on ne sait pas trop quoi croire, mis à part des sites vraiment spécialisés. » (P42-43)*

*« […] il faut vérifier les infos soit par un spécialiste soit autre. Mais ça ne peut pas être la seule source d’informations… » (P12-44)*

*« On peut parler de toutes les sources d’information sur l’alimentation pendant la grossesse […] mais au final la décision c‘est nous qui la prenons ! »(P53-45)*

*« On le sait déjà instinctivement, on sait qu’il ne faut pas manger trop gras, trop sucré, qu’il faut manger des légumes, les grandes lignes en fait on les connaît. » (P12-46)*
