# Supplementary material for: Concerns, attitudes, beliefs and information seeking practices with respect to nutrition-related issues: a qualitative study in French pregnant women
Source: BMC Pregnancy Childbirth. 2016 Oct 12;16:306. doi: 10.1186/s12884-016-1078-6 (PMC5059968; doi:10.1186/s12884-016-1078-6)
Supplement: Additional file 3: — Identification number, session, recruitment place, trimester of pregnancy and parity of each participant. (DOCX 15 kb) [file 12884_2016_1078_MOESM3_ESM.docx]

**Additional file 3.** Identification number, session, recruitment place, trimester of pregnancy and parity of each participant

| **Identification number** | **Session** | **Recruitment place** | **Age** | **Trimester of pregnancy** | **Parity** |
| --- | --- | --- | --- | --- | --- |
| P11 | 1 | Aix-en-Provence | 36 | 3rd | Multiparous |
| P12 | 1 | Aix-en-Provence | 34 | 3rd | Multiparous |
| P13 | 1 | Aix-en-Provence | 40 | 3rd | Multiparous |
| P14 | 1 | Aix-en-Provence | 31 | 2nd | Primiparous |
| P15 | 1 | Aix-en-Provence | 39 | 3rd | Multiparous |
| P16 | 1 | Aix-en-Provence | 31 | 3rd | Primiparous |
| P17 | 1 | Aix-en-Provence | 36 | 2nd | Multiparous |
| P21 | 2 | Aix-en-Provence | 24 | 3rd | Primiparous |
| P22 | 2 | Aix-en-Provence | 30 | 1st | Multiparous |
| P23 | 2 | Aix-en-Provence | 32 | 3rd | Primiparous |
| P24 | 2 | Aix-en-Provence | 28 | 3rd | Primiparous |
| P25 | 2 | Aix-en-Provence | 33 | 1st | Multiparous |
| P26 | 2 | Aix-en-Provence | 21 | 2nd | Primiparous |
| P31 | 3 | Paris | 32 | 2nd | Multiparous |
| P32 | 3 | Paris | 30 | 2nd | Multiparous |
| P33 | 3 | Paris | 36 | 2nd | Primiparous |
| P34 | 3 | Paris | 28 | 2nd | Multiparous |
| P35 | 3 | Paris | 36 | 3rd | Primiparous |
| P41 | 4 | Paris | 28 | 2nd | Multiparous |
| P42 | 4 | Paris | 24 | 2nd | Multiparous |
| P51 | 5 | Paris | 26 | 2nd | Multiparous |
| P52 | 5 | Paris | 27 | 2nd | Primiparous |
| P53 | 5 | Paris | 31 | 2nd | Primiparous |
| P54 | 5 | Paris | 27 | 2nd | Multiparous |
| P55 | 5 | Paris | 23 | 2nd | Primiparous |
| P56 | 5 | Paris | 32 | 2nd | Primiparous |
| P57 | 5 | Paris | 28 | 1st | Multiparous |
| P61 | 6 | Paris | 28 | 2nd | Primiparous |
| P62 | 6 | Paris | 29 | 1st | Multiparous |
| P63 | 6 | Paris | 28 | 2nd | Primiparous |
| P64 | 6 | Paris | 32 | 2nd | Primiparous |
| P65 | 6 | Paris | 33 | 3rd | Primiparous |
| P66 | 6 | Paris | 26 | 3rd | Primiparous |
| P67 | 6 | Paris | 33 | 1st | Multiparous |
| P71 | 7 | Paris | 30 | 2nd | Primiparous |
| P72 | 7 | Paris | 31 | 3rd | Multiparous |
| P73 | 7 | Paris | 27 | 3rd | Multiparous |
| P74 | 7 | Paris | 35 | 3rd | Multiparous |
| P75 | 7 | Paris | 31 | 3rd | Primiparous |
| P76 | 7 | Paris | 32 | 2nd | Primiparous |
